# Sequence Searcher: A Java tool to perform regular expression and fuzzy searches of multiple DNA and protein sequences

**DOI:** 10.1186/1756-0500-2-14

**Published:** 2009-01-30

**Authors:** Francesco Marass, Chris Upton

**Affiliations:** 1 Biochemistry and Microbiology, University of Victoria, Victoria, BC V8W 3P6, Canada

## Abstract

**Background:**

Many sequence-searching tools have limiting factors for their use. For example, they may be platform specific, enforce restrictive size limits and sequences to be searched, or only allow searches of one of DNA or protein.

**Findings:**

We present an easy-to-use, fast, platform-independent tool to search for amino acid or nucleotide patterns within one or many protein or nucleic acid sequences. The user can choose to search for *regular expressions *or perform a *fuzzy search *in which a particular number of errors is accepted during matching of a sequence. Positions of mismatches in fuzzy searches are displayed graphically the user.

**Conclusion:**

SeqS provides an improved feature set and functions as a stand-alone tool or could be integrated into other bioinformatics platforms.

## Background

Searching for specific patterns in protein and DNA sequences is a common analysis performed by molecular biologists. Detection of restriction enzyme cleavage sites in DNA sequences was an early use of this pattern matching process. Later, as the protein databases grew, the PROSITE motif database was developed [1]. These protein motifs are written as regular expressions that capture the variability within a consensus sequence from a short, highly conserved region in a multiple alignment. As the volume and diversity of genomic information grows, it is necessary to modify PROSITE patterns to allow them to match more diverse homologs. Searching through genomic sequences for conserved nucleotide patterns such as transcription factor binding sites is another use for this type of analysis. Large-scale sequencing has lead to some automated bioinformatics analyses, but pattern searching is such a common "hands on" interactive procedure that we have developed an easy-to-use tool, Sequence Searcher (SeqS), that supports searching for user-specified patterns in multiple protein and nucleotide sequences. SeqS has been integrated into several of the Viral Bioinformatics Resource Center tools and can therefore read sequences directly out of the VOCs database [2], but it also function as a stand-alone program with the ability to manage sequences much larger than viral genomes.

### Algorithms

We implemented a brute-force fuzzy search algorithm and made use of the Jakarta ORO libraries [3] for Perl-like regular expressions. To speed up the searches and reduce memory requirements, an algorithm to reverse both fuzzy patterns and regular expressions was developed. In a search on DNA, SeqS first searches the top strand (the sequence itself), and then the bottom strand. However, rather than creating a duplicate (the bottom strand) of the nucleotide sequence, which is expensive in terms of memory, SeqS reverses and complements the query and searches the top strand again.

### Implementation

**Figure 1:**
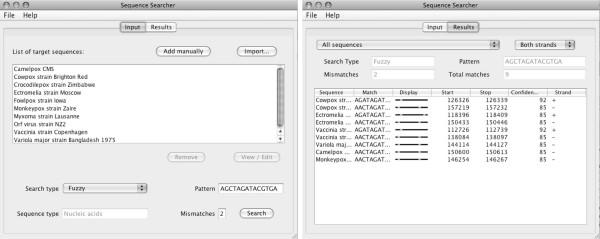
**SeqS user interface**. Left side: Input panel, 10 poxvirus genomes searched with a fuzzy pattern; right side: Results panel, showing positions of mismatches within matched pattern.

In the development of SeqS, our goal was to produce an intuitive interface with easily understood output. The stand-alone interface of SeqS is simple and consists of two panels, accessed by the *input *and *results *tabs (Figure 1). In the *input *panel, users can import sequences to the workspace, set the search parameters and initiate the search. SeqS accepts one or many sequences (FASTA format) as input, either *imported *from a text file or directly pasted into a text box (*add manually)*. Sequence names are shown in the *List of target sequences *box and the program uses selected sequences, or all sequences if none are selected. Sequence Searcher recognises ambiguity codes for amino acids and nucleotides as described by IUPAC [4], and tries to determine if a sequence contains nucleic acid or amino acids. SeqS supports two search types: *regular expression *and *fuzzy*. Although a *regular expression *may describe variation (AAA [CG], AAA followed by C or G), these searches are always exact and in this example only match patterns specified by the user (AAAC or AAAG). Supported *regular expression *patterns are *characters *(such as A and C), *character sets *(such as [AG]), *quantifiers *(such as +, ?, *, {*n*, *m*}) and *back references *(such as (NNNN)\1). The pattern AAA [^C] [GT].{1,10}(AT)\1 translates to AAA, anything but C, then G or T, anything 1–10 times, AT repeated twice. A useful feature of regular expressions is the ability to allow variable space between 2 patterns. In contrast, a *fuzzy *search allows a specified number of mismatches within the pattern; e.g. ATGATAGT with 3 mismatches. However, the *fuzzy *search also supports a basic syntax similar to regular expressions to allow even more flexible pattern recognition; it is possible to match one of some characters by listing them within square brackets ([AG] matches either A or G) and it is also possible to exclude characters by placing them within braces ({AG} matches neither A nor G). SeqS allows the number of mismatches to be 0–40% of the pattern length; this prevents the search from taking too long and returning too many imprecise matches.

Upon completion of a search, the *results *panel is presented, showing the data in tabular format (Figure 1). It is possible to sort the table by any data column using the column header (sequence, match, match start, match stop, confidence, strand) as well as filter the results set by sequence and by strand using drop-down menus. The search parameters are also reported. To facilitate interpretation of *fuzzy *search results, an additional graphical representation of the match is included in the form of a multi-coloured line that is divided into a series of segments numbering equal to the length of the pattern match. A segment is coloured green if a character match is exact, orange if it matches an ambiguity character and red in the case of a mismatch. The user can choose to save the results (all or selected rows) to a tab-delimited text file that is easily imported into a spreadsheet for further analysis.

SeqS is limited by the amount of memory dedicated to the Java Virtual Machine and may run out of memory during import of sequences or performing a search. However, for most practical work with viral and bacterial genomes, these limits are of no consequence. For example, Table 1 shows a series of searches performed with a set of sequences totalling 170 Mb that each took 4 s or less (iMac with 2.4 GHz Core 2 Duo and 4 GB RAM). SeqS imports the test sequences at approximately 1 Mb/s.

**Table 1:** Examples of SeqS searches on DNA sequences totalling 170 MB. All searches took less than 4 seconds

Fuzzy Search			Regular Expression Search	
Search Pattern	No. of mismatches	No. of hits	Search Pattern	No. of hits

ACGTACGTA	0	44	ACGTACGT	216

ACGTACGTA	1	1808	ACGACGTA	108

ACGTACGTA	2	22124	ACGACGT	244

ACGTACGTACGT	1	24	ACGA [CGTA]GT	948

ACGTACGTACGT	2	672	ACGA.G{1,3}T	1272

ACGTACGTACGTACGT	3	80	ACGA.{1,50}G{1,3}T	23991

## Conclusion

SeqS is a versatile tool that can be used as a stand-alone program or easily incorporated into more complex bioinformatics workbenches. It provides the ability to search multiple sequences in a single run with regular expressions or fuzzy patterns. Results are displayed in sortable tables and graphics are used to show fuzzy matches. To enable viewing of results with genome annotations, the core of SeqS has been incorporated in to the Viral Genome Organizer [5] and Base-By-Base [6] tools that can read GenBank files.

## Availability and requirements

**Project name: **Sequence Searcher (SeqS)

**Project homepage: **

**Operating system: **Platform independent

**Programming language: **Java

**Other requirements: **Java 1.4 or higher; Java Web Start

**License: **SeqS is distributed under the Open Software License.

**Any restrictions to use by non-academics: **None

## Competing interests

The authors declare that they have no competing interests.

## Authors' contributions

CU conceived the idea and specifications; FM developed the code; both authors tested the tool and contributed to writing the manuscript.
